# Efficacy of aflibercept in vitrectomized vs. non-vitrectomized eyes with diabetic macular edema: a prospective study

**DOI:** 10.1186/s40942-025-00778-y

**Published:** 2025-12-09

**Authors:** Géza Thury, Nóra Baranyi, Ferenc Rárosi, Rebeka Héjja, Attila Kovács

**Affiliations:** 1 Department of Ophthalmology, University of Szeged, Korányi Fasor 10-11, Szeged, 6720 Hungary; 2 Department of Biostatistics, University of Szeged, Szeged, Hungary

**Keywords:** Aflibercept, Vitrectomy, Diabetic macular edema, Retina

## Abstract

**Background:**

Diabetic macular edema (DME) is a leading cause of visual impairment in patients with diabetes. Intravitreal anti-vascular endothelial growth factor (VEGF) agents are the first-line treatment for DME, with aflibercept having demonstrated favorable outcomes in comparative studies. However, data on the efficacy of anti-VEGF therapy in vitrectomized eyes are inconclusive. Corticosteroids, specifically dexamethasone (DEX) implant, may be used for refractory cases. This study aimed to evaluate the efficacy of intravitreal aflibercept (IVA) in patients with DME with or without prior pars plana vitrectomy (PPV) and to evaluate the outcomes of DEX implantation in refractory cases.

**Methods:**

This prospective single-center study included 46 eyes with center-involved DME. Eyes were divided into PPV and intact vitreous body (non-PPV) groups. All patients received IVA injections following the DRCR.net Protocol T. After six monthly injections, eyes refractory to IVA treatment were switched to DEX implant (refractory group). In the non-refractory group IVA injections were administered as needed (PRN) until month 12. The best-corrected visual acuity (BCVA) and central subfield thickness (CSFT) were measured at baseline; at 1 week; monthly through month 6 in all eyes; monthly through month 12 in the non-refractory group; and at 2 months after DEX implantation in the refractory group.

**Results:**

There were 23 eyes each in the PPV and non-PPV groups. Overall, 13 (28.3%) eyes were refractory to IVA (8 PPV, 5 non-PPV; *p* > 0.05). The median number of IVA injections among PPV and non-PPV eyes in the non-refractory group over 12 months showed no significant difference (PPV: 10; non-PPV: 9.5; *p* > 0.05). Both groups showed significant improvement in BCVA (PPV: +7.0 letters; non-PPV: +11.1 letters; both *p* < 0.01) and CSFT (PPV: − 182.5 μm; non-PPV: − 190.4 μm; both *p* < 0.01) at 12 months. In refractory cases, DEX implantation resulted in a significant CSFT reduction (–259.1 μm, *p* < 0.01) but not BCVA improvement.

**Conclusion:**

IVA is effective for DME regardless of vitreous status, with similar efficacy and treatment frequency in vitrectomized and non-vitrectomized eyes. DEX implantation produces anatomical benefits in IVA-refractory cases, although visual gains are limited.

## Background

Diabetic macular edema (DME) is a major cause of visual impairment in patients with diabetes. DME can develop at any stage of diabetic retinopathy (DR), and its prevalence is increasing [1–3].

The first-line treatment of center-involved DME (CI-DME) includes intravitreal anti- vascular endothelial growth factor (VEGF) agents [1, 2, 4–6]. Intravitreal corticosteroids, specifically dexamethasone (DEX) implants, are second-line options if 3–6 anti-VEGF injections produce unsatisfactory results [1, 2, 7, 8].

The commonly used anti-VEGF agents in CI-DME are aflibercept, bevacizumab, and ranibizumab [1, 5, 9, 10]. The DRCR.net Protocol T compared the efficacy and safety of these agents [5, 9, 10] and reported that while all improved visual outcomes, aflibercept resulted in greater visual improvement in patients with more severe (< 69 Early Treatment Diabetic Retinopathy Study (ETDRS) letters) vision loss at baseline (BL) [1–3, 5, 10]. High-dose aflibercept (8 mg) or other anti-VEGF agents, such as faricimab or brolucizumab, for DME have been evaluated in company-sponsored clinical trials, but no independent studies have examined them using the same therapeutic regimen as that in Protocol T [1, 2, 11–13].

Due to its safety profile and range of indications, the use of pars plana vitrectomy (PPV) is increasing in developed countries [14–16]. However, no real-world studies have evaluated the pharmacodynamics and pharmacokinetics of anti-VEGF agents in vitrectomized eyes. Most data in the literature come from animal models (e.g., macaque or rabbit eyes) wherein the properties of the vitreous may not be similar with those in human eyes [17]. Furthermore, studies on human eyes are mostly retrospective and have a diversified methodology, small number of patients, short follow-up duration, and imbalanced BL characteristics between the different vitreous condition groups or do not have a control group at all [17–19].

Therefore, this prospective, one-year study was designed to evaluate the efficacy of IVA injections for DME in eyes with different vitreous conditions, including vitrectomized and non-vitrectomized eyes. A secondary objective was to investigate the effectiveness of DEX implants in cases refractory to IVA therapy.

## Methods

This prospective, single-center, single-blind, interventional cohort study was conducted from March 2023 to March 2025. The Ethics Committee of the University of Szeged reviewed and approved the trial (Protocol no. NH_SiOP-001, Reference no. 168/2022-SZTE RKEB). This study was conducted in accordance with the tenets of the Declaration of Helsinki.

### Patient selection

Patients with type 1 or type 2 diabetes mellitus aged > 18 years, with a hemoglobin A1c level < 12% in the last 3 months, and without modifications to their diabetic medication in the prior 3 months were enrolled.

After obtaining informed consent, patients with CI-DME requiring intravitreal anti-VEGF injections were classified into the PPV and non-PPV groups according to their vitreous condition. The PPV group included previously vitrectomized eyes performed more than 3 months before enrollment without silicone oil (SiO) tamponade or had undergone SiO removal > 3 months before study participation. The non-PPV group included eyes with an intact vitreous body. The inclusion criteria for CI-DME requiring intravitreal anti-VEGF injections were a best-corrected visual acuity (BCVA) ranging from 24 to 78 letters based on the standardized Early Treatment Diabetic Retinopathy Study (ETDRS) chart and a central subfield thickness (CSFT) ≥ 320 μm regardless of an unaltered vitreous or prior vitrectomy. The CSFT was measured using spectral-domain optical coherence tomography (SD-OCT) (Heidelberg Engineering GmbH, Heidelberg, Germany) (pattern size of 5.6 × 5.6 mm, 20°× 20° was applied with 49 B-scans, using the “follow-up” mode). The BCVA examiner and OCT analyzer were both blinded to the vitreous condition of the eyes. The administered intravitreal anti-VEGF agent was aflibercept (Eylea; Regeneron Pharmaceuticals, Inc. [Tarrytown, NY, USA]) (2 mg/0.05 mL) in all cases.

Being treatment-naïve to DME treatment was not a prerequisite for participation in the study; however non-treatment-naïve eyes with DME had to fulfill the following requirements:

- At least 12 weeks have passed since the last administration of anti-VEGF and initiation of this study.
- At least 12 weeks have passed since the previous macular laser or panretinal photocoagulation treatment and initiation of this study.
- At least 4 months must have passed since the last intravitreal administration of triamcinolone acetonide, or at least 6 months since the last DEX implantation, and initiation of this study.

### Treatment protocol

Patient visits and treatments were performed in accordance with Protocol T, with all assessments conducted following this clinical protocol. BCVA evaluations and CSFT measurements were performed on every visit. The initial (BL) visit also included the first administration of IVA. One week after, a follow-up was conducted without IVA administration to detect early changes in BCVA and CSFT. Subsequent visits from month 1 (M1) to M5 included IVA administration except under the following conditions: the BCVA was 20/20 or better and CSFT was < 320 μm, and no improvement (an increase of 5 letters or a 10% decrease in CSFT) or deterioration (a decrease of 5 letters or a 10% increase in CSFT) was observed following the last two injections. Upon fulfillment of these criteria, IVA injections were discontinued prior to the completion of the six mandatory injections.

We also established the rescue treatment criteria, which were the Protocol T and Protocol V criteria combined, However, instead of employing the macular laser, IVA treatment was shifted to DEX implantation (Ozurdex; Allergan, Inc. [Irvine, CA, USA]) (0.7 mg) within the initial six visits, if:

- following three injections, there was a loss of ≥ 10 letters throughout two injection visits relative to the BL [4]; and.
- CSFT was ≥ 320 μm [5, 10]; and.
- DME was present, and the examining physician concluded that DME was responsible for vision loss [5, 10].

At M6, the necessity for refractory treatment was determined. In refractory cases, IVA injections were discontinued, and intravitreal DEX was implanted. Although we followed the refractory criteria of Busch et al. [16], we implanted DEX after six monthly IVA injections to accommodate eyes that react slowly to treatment. The refractory criteria were met if:

- There was a ≤ 20% CSFT reduction compared to BL; or.
- The BCVA gain was ≤ 5 letters compared to BL.

Unless patients met the rescue or refractory criteria, we conducted monthly visits from M6 to M12 and treated the patients in a PRN basis according to Protocol T. IVA treatment was unnecessary if eyes showed stability, and no improvement or deterioration occurred over the last two injections; otherwise, IVA was administered.

BCVA and CSFT were assessed in all patients at BL, at 1 week, and then monthly through M6. In the non-refractory group, these parameters were further evaluated monthly from M6 to M12. In the refractory group, BCVA and CSFT were reassessed 2 months after DEX implantation.

### Outcome measures and statistical analysis

The primary outcomes of the study were to determine the distribution of IVA refractory and non-refractory cases at M6 according to vitreous cavity status, and to evaluate the number of IVA injections required in non-refractory eyes during the 12-month follow-up, together with their BCVA and CSFT outcomes. The secondary outcome was to evaluate the effect of DEX implants in refractory eyes by assessing changes in BCVA and CSFT two months after implantation.

Based on the data from BL to M6, we attempted to determine a predictive factor for the early identification of eyes refractory to IVA treatment and evaluated its specificity and sensitivity.

Continuous data were summarized as mean ± SD for approximately symmetric distributions, or as median with quartiles for skewed distributions. Categorical variables were presented as frequencies (number of cases) and relative frequencies (percentages).

Baseline characteristics between the two study arms were compared using Welch’s independent samples t-test or the Mann–Whitney U-test for continuous variables, and the chi-square test for independence or Fisher’s exact test for categorical variables. To assess the effects of time and study arm, a two-way repeated measures analysis of variance (ANOVA) was performed. Post-hoc analyses for significant main effects were conducted using the least significant difference (LSD) method.

Further analysis of M12 CSFT and BCVA values was performed using a multivariate analysis of covariance (ANCOVA) to assess differences between study groups. Three categorical between-subject factors were included: vitreous status, DR severity (binary: proliferative DR and nonproliferative DR) and treatment naïve status. BL BCVA was entered as a covariate to adjust for initial visual function.

The model incorporated both main effects and the interaction between vitreous status, DR severity and treatment naïve status. Estimated marginal means were calculated for each factor, and pairwise comparisons were performed using the LSD adjustment.

The reduction in CSFT from BL to M1 was further evaluated with receiver operating characteristic (ROC) curve analysis. The area under the ROC curve (AUC) was reported with its 95% confidence interval. The optimal cut-off value was determined by maximizing the Youden-index. P-values *p* < 0.05 were regarded as statistically significant. All analyses were performed using IBM SPSS Statistics, version 29.0.0.0 (Build 241).

Sample size estimation was based on the Mann–Whitney U-test for independent samples. The test power was set at 80%, with an assumed effect size of d = 1.2. The distribution family “min Are” was chosen, and the significance level was set at 5%. This yielded a required total sample size of 28 participants (14 per group). Sample size calculation was conducted with G*Power software (version 3.1.9.7, University of Düsseldorf).

## Results

### Study participants

A total of 49 eyes of 49 patients with DME were enrolled in the study; 3 were excluded due to missed visits, resulting in 46 eyes in the final analysis. Of these, 23 eyes had undergone PPV (PPV group), and 23 eyes had an unaltered vitreous body (non-PPV group). Indications for PPV included vitreous hemorrhage (65.2%), epiretinal membrane (17.4%), tractional retinal detachment (8.7%), vitreomacular traction (4.4%), and complicated cataract surgery (4.4%). The BL characteristics are presented in Table 1.

**Table 1:** Baseline characteristics of the two study arms (NPDR: non-proliferative diabetic retinopathy, PDR: proliferative diabetic retinopathy, IVI: intravitreal injection, DME: diabetic macular edema, anti-VEGF: anti-vascular endothelial growth factor)

	Vitrectomized eyes (*n* = 23)	Non-vitrectomized eyes (*n* = 23)	*p* value
Age (years mean ± SD)	60.9 ± 14.4	64.1 ± 7.4	0.335 (Welch independent samples t-test)
Gender (male: female, number of cases)	12:11	11:12	0.768 (Chi square test for independence)
HbA1c level (%) (mean ± SD, Q1, med, Q3)	7.1 ± 1.4 Q1 = 6.4 med = 7.3 Q3 = 8.1	7.4 ± 1.4 Q1 = 6.33 med = 7.3 Q3 = 7.9	0.929 (Mann-Whitney u-test)
Type of diabetes (type1:type2, number of cases)	2:21	1:22	1 (Fisher’s exact test)
Severity of DR (n) (mild NPDR: moderate NPDR: severe NPDR: PDR, number of cases)	1:1:1:20	5:9:8:1	*p* < 0.01 (Fisher’s exact test)
Treatment naïve to prior IVI (n)	1	14	*p* < 0.01 (Fisher’s exact test)
Previous DME treatment (%) • anti-VEGF • corticosteroid • macular laser	82.6% 34.8% 13%	21.7% 26.1% 21.7%	

Before study participation, the previous DME treatment options were heterogenous, with most patients receiving combined therapy such as intravitreal anti-VEGF injections (bevacizumab or aflibercept), corticosteroids (peribulbar triamcinolone or intravitreal DEX implant), macular laser, or a combination of these options.

### Rescue treatment and IVA-refractory cases

None of the 46 eyes required rescue treatment. At M6, 13 eyes met the criteria for refractory treatment. After six IVA injections, eight and five eyes from the PPV and non-PPV groups, respectively, received DEX implants. Notably, the vitreous status between both groups was not significantly different.

### Frequency of IVA injections

The non-refractory group (*n* = 33; 15 PPV, 18 non-PPV) received IVA injections alone during the 1-year follow-up period. The average number of IVA injections at M12 was 9.4 ± 2.1 (median = 10) in the PPV group and 8.8 ± 1.7 (median = 9.5) in the non-PPV group; the difference was not significant.

### Effect of treatment on visual acuity (VA)

**Fig. 1:**
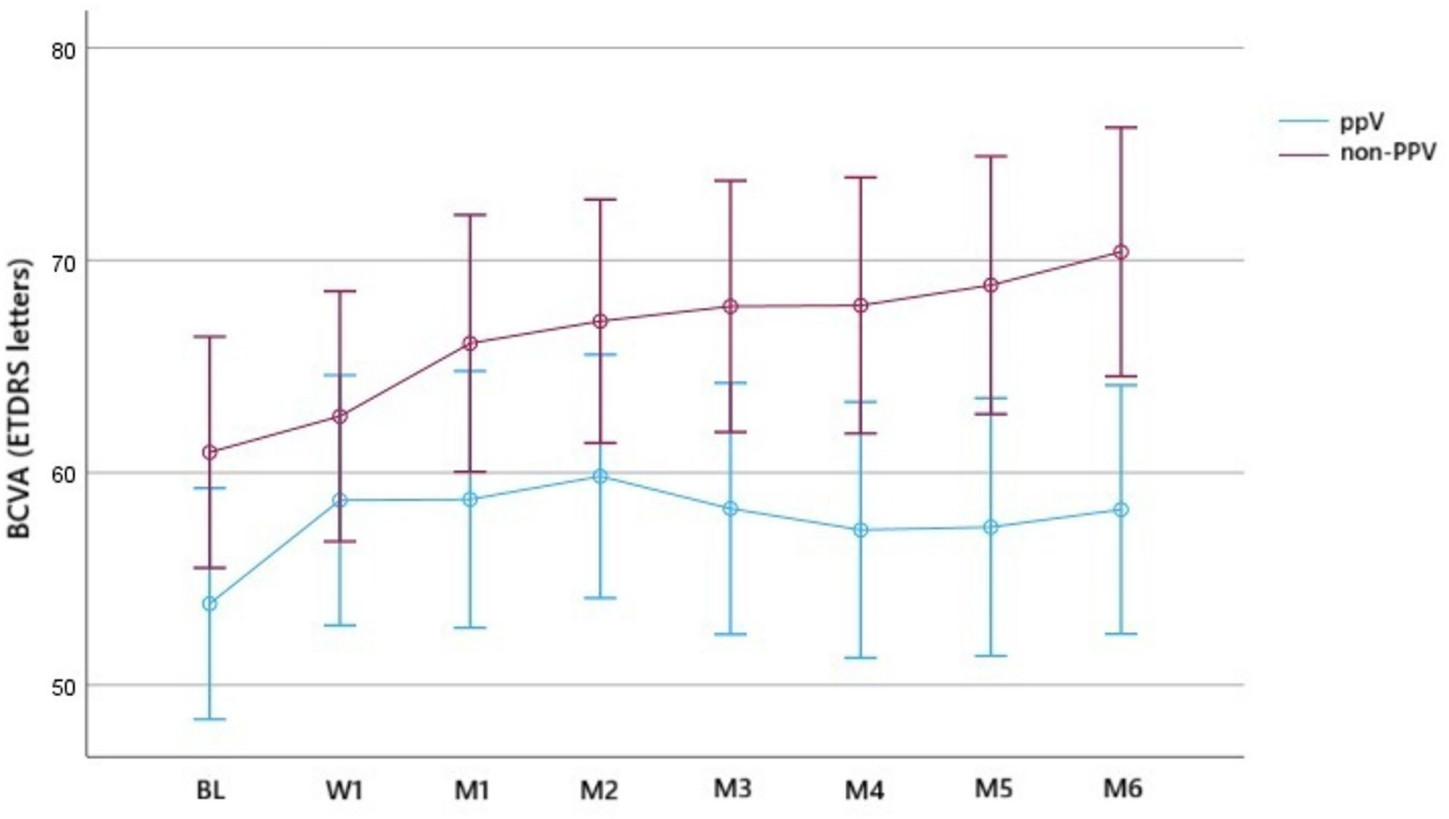
Time series plot showing the change in best-corrected visual acuity (BCVA) in the vitrectomized (PPV) and non-vitrectomized (non-PPV) groups until month 6

In all eyes, the mean BL BCVA was 53.8 ± 14.3 letters in the PPV group and 61.0 ± 11.5 letters in the non-PPV group (*p* = 0.069). At M6, the mean BCVA was 58.3 ± 17.1 letters in the PPV group and 70.4 ± 9.9 letters in the non-PPV group (*p* < 0.01). The mean BCVA gain from BL to M6 was 4.4 ± 1.3 letters and 9.4 ± 1.3 letters in the PPV group and non-PPV groups, respectively (both *p* < 0.01) (Fig. 1).

**Fig. 2:**
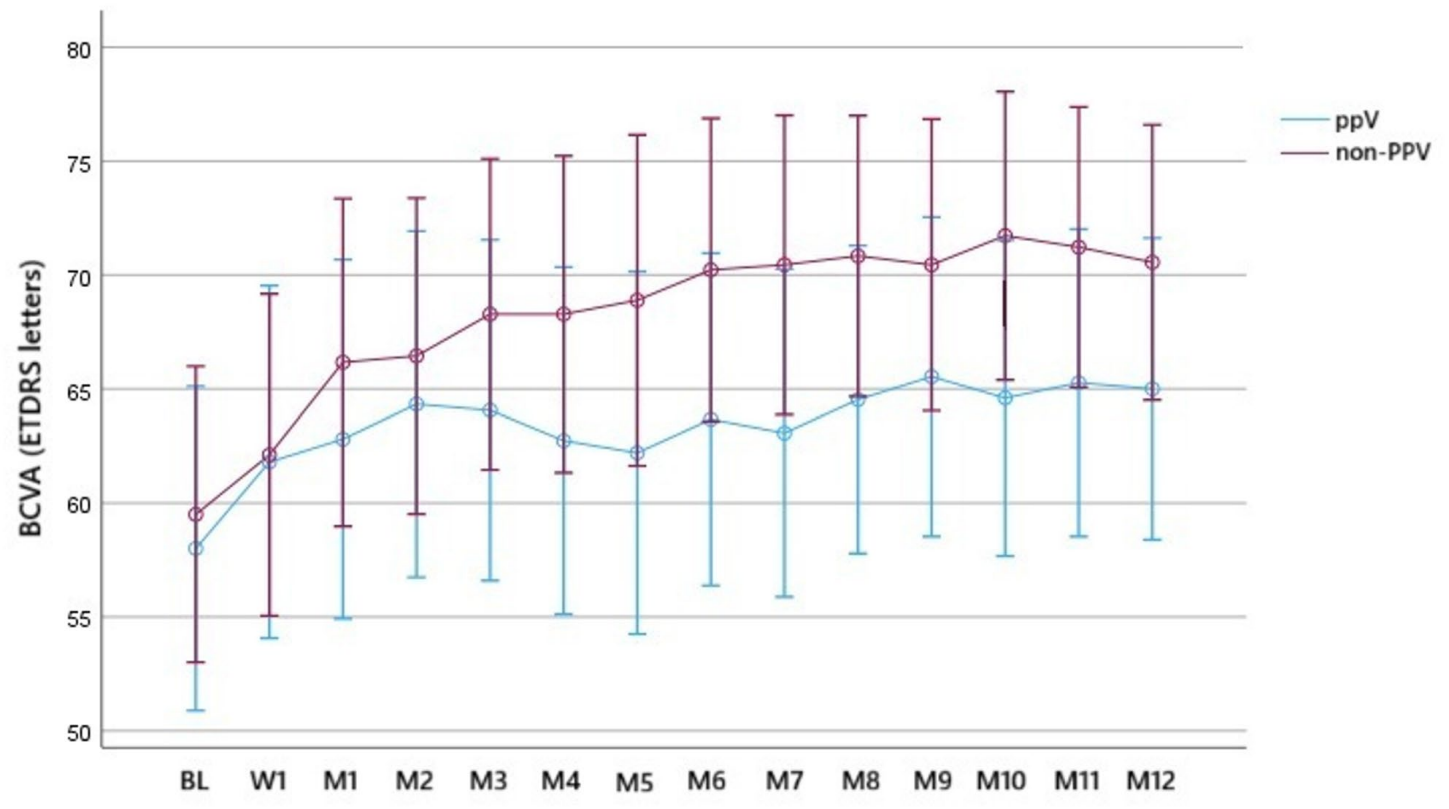
Time series plot showing the change in best-corrected visual acuity (BCVA) in non-refractory cases until month 12 according to their vitreous status (vitrectomized, PPV; non-vitrectomized, non-PPV)

Among non-refractory cases that received IVA injections alone, the mean BL BCVA was 58.0 ± 14.9 letters in the PPV group and 59.5 ± 12.3 letters in the non-PPV group. At M6, the mean BCVA was 63.7 ± 16.6 and 70.2 ± 11.1 letters in the PPV and in the non-PPV groups, respectively. At M12, the mean BCVA was 65.0 ± 15.4 and 70.6 ± 9.7 letters in the PPV and non-PPV groups, respectively. Regarding the vitreous status, there was no significant difference at any time point. The mean BCVA gain from BL to M6 was 5.7 ± 1.6 letters in the PPV group and 10.7 ± 1.5 letters in the non-PPV group (both *p* < 0.01), while from BL to M12, it was 7.0 ± 2.1 letters in the PPV group and 11.1 ± 2 letters in the non-PPV group (both *p* < 0.01). Meanwhile, the mean BCVA gain from M6 to M12 was 1.3 ± 1.7 letters in the PPV group and 0.3 ± 1.7 letters in the non-PPV group; the differences were not significant (Fig. 2).

### Effects of treatment on retinal thickening

**Fig. 3:**
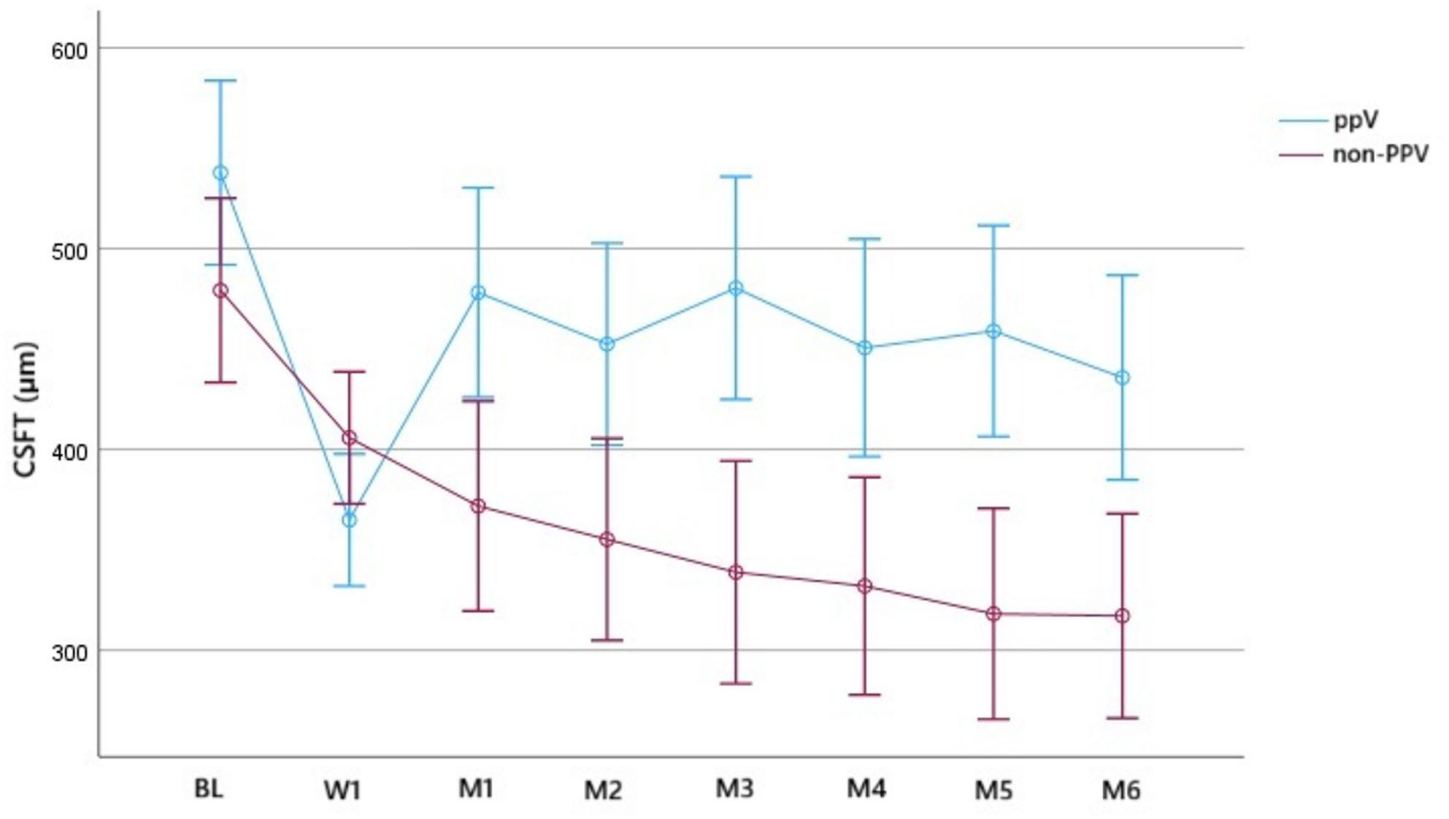
Time series plot showing the central subfield thickness (CSFT) in the vitrectomized (PPV) and non-vitrectomized (non-PPV) groups until month 6

In all eyes, the mean CSFT at BL was 537.8 ± 128.1 μm in the PPV group and 479.2 ± 86.1 μm in the non-PPV group (*p* = 0.075). At M6, the mean CSFT was 435.8 ± 153.5 μm in the PPV group and 317.1 ± 76.2 μm in the non-PPV group (*p* < 0.01). The mean CSFT reduction from BL to M6 was 102 ± 23.4 μm and 162.1 ± 23.4 μm in the PPV and non-PPV groups, respectively (both *p* < 0.01) (Fig. 3).

**Fig. 4:**
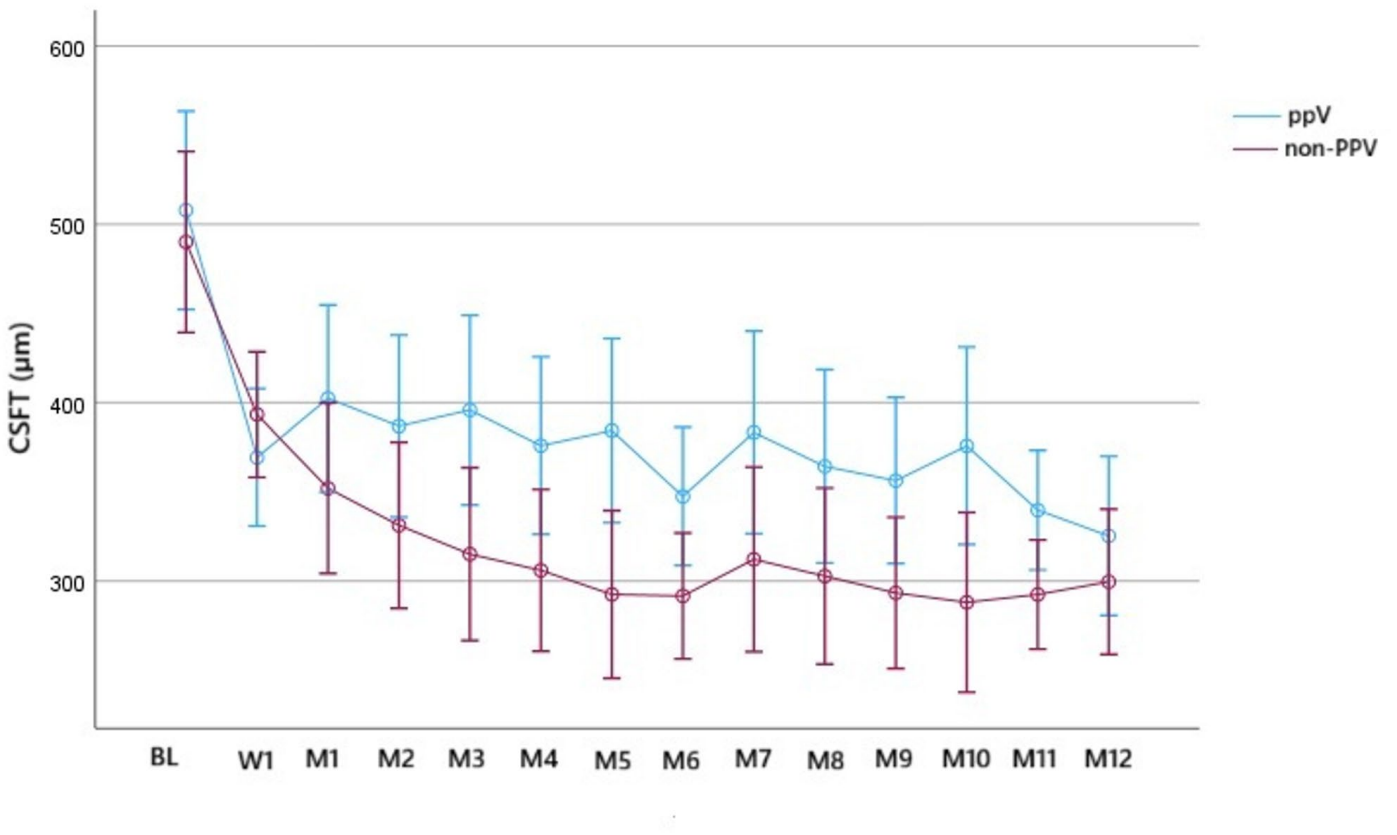
Time series plot showing the central subfield thickness change (CSFT) in non-refractory cases until month 12 according to their vitreous status (vitrectomized, PPV; non-vitrectomized, non-PPV)

Among non-refractory cases that received IVA injections alone, the mean CSFT at BL was 507.9 ± 122.4 μm and 490.1 ± 89.3 μm in the PPV and non-PPV groups, respectively. The mean CSFT was 347.5 ± 86.8 μm and 291.7 ± 60.3 μm at M6 and 325.3 ± 66.5 μm and 299.6 ± 97.3 μm at M12 in the PPV and non-PPV groups, respectively. There was significant difference only at M6 (*p* < 0.05). The mean CSFT reduction from BL to M6 was 160.4 ± 23.2 μm in the PPV group and 198.4 ± 21.1 μm in the non-PPV group (both *p* < 0.01), while from BL to M12, it was 182.5 ± 29.2 μm in the PPV group and 190.4 ± 26.6 μm in the non-PPV group (both *p* < 0.01). Meanwhile, the mean CSFT reduction from M6 to M12 was 22.1 ± 15 μm in the PPV group and it was actually thickening (7.9 ± 13.7 μm in the non-PPV group (Fig. 4).

### Multivariate ANCOVA results of BL characteristics

M12 multivariate results of BCVA: The ANCOVA model examining BCVA at M12 revealed BL BCVA in non-refractory eyes as the dominant predictor (*p* < 0.001), indicating that initial visual acuity of non-refractory eyes strongly determines visual outcomes at M12. In contrast, neither vitreous status (*p* = 0.201) nor DR severity (*p* = 0.183) showed significant main effects after adjustment for BL BCVA, and both exhibited small effect sizes. All interaction terms, including the vitreous status × DR severity interaction and those involving treatment-naïve status, were non-significant (*p* > 0.70), suggesting that these factors do not modify the effect on BCVA at M12.

M12 multivariate results of CSFT: The ANCOVA model assessing CSFT at M12, indicated that the included predictors did not meaningfully explain M12 CSFT. BL BCVA in non-refractory eyes was not a significant covariate (*p* = 0.542), suggesting no association between initial visual acuity of non-refractory eyes and CSFT at M12. None of the main effects reached statistical significance: vitreous status (*p* = 0.243), treatment naïve status (*p* = 0.124), and DR severity (*p* = 0.879). All showed small or negligible effect sizes. Likewise, none of the interaction terms were significant (*p* > 0.18), providing no evidence that combinations of these factors influenced CSFT at M12.

### Effect of DEX implants on VA and retinal thickening

In the 13 eyes that met the refractory criteria, the mean BCVA was 56.9 ± 15.7 letters after 6 months of IVA injections, whereas the mean BCVA 2 months after DEX implantation improved to 59.7 ± 14.6 letters (*p* = 0.091). The mean CSFT was 527.3 ± 130.1 μm after 6 months of IVA injections, whereas the mean CSFT 2 months after DEX implantation was 268.2 ± 66.2 μm (*p* < 0.01).

### Predictive factor

We examined the CSFT reduction from BL to M1 using ROC analysis, revealing an area under the ROC of 0.895 (95% CI: 0.784, 1.000), indicating very good predictive performance. The threshold (cut-off value) was selected because a small number of false negative cases was desirable to detect refractory cases with high sensitivity (probability). If the CSFT reduction from BL to M1 was < 10.5%, that eye is expected to be refractory to IVA treatment, with a specificity of 72.7% and sensitivity of 92.3%.

### Safety

No serious local (e.g., endophthalmitis) or systemic adverse events were noted during the 1-year follow-up period.

## Discussion

It is hypothesized that drug clearance is increased in vitrectomized eyes, resulting in reduced drug effectiveness. However, previous studies have demonstrated mixed results, with some reporting worse outcomes and faster drug clearance in vitrectomized eyes and others finding no significant difference [17–20]. In larger clinical trials on aflibercept (e.g., DRCR. net Protocol T or VISTA/VIVID), the vitreous status was not indicated [6, 10], while studies on the pharmacodynamics and pharmacokinetics of anti-VEGF agents in vitrectomized eyes has limitations (e.g., small sample size, short follow-up period, no control group, etc.).

Regarding the efficacy of IVA, there was no significant difference in the number of IVA-refractory eyes between vitrectomized and non-vitrectomized eyes, and rescue treatment was not necessary in either group. We also examined the frequency of IVA injections in non-refractory cases based on the vitreous status, showing that the median number of IVA injections after the 1-year follow-up period was 10 in the PPV group and 9.5 in the non-PPV group with no significant difference. These results suggest that the vitreous condition may not impact the effectiveness of aflibercept in DME. Furthermore, vitrectomized eyes may not require more frequent injections to maintain the efficacy of aflibercept. Regarding the functional and anatomical outcomes, CSFT values were more variable in the PPV group compared to the non-PPV group (Figs. 3 and 4); this is in contrast to the BCVA values (Figs. 1 and 2). To determine the early response to IVA, BCVA and CSFT were assessed 1 week (W1) after IVA administration, revealing that the mean W1 CSFT was lower in the PPV group than in the non-PPV group, but the difference was not significant However, at M1, eyes in the PPV group had thicker retinas than those in the non-PPV group, but the difference was not significant (Figs. 3 and 4). Conversely, the mean BCVA values did not exhibit these changes (Figs. 1 and 2). These findings may be attributed to the altered drug clearance in vitrectomized eyes; however, the 1-year results indicate that the pharmacodynamics and pharmacokinetics of aflibercept are not based on the vitreous condition. Starr et al. reported that a greater fluctuation in macular edema on OCT after two years is associated with worse functional outcomes after analyzing the results of Protocol T and V [21]. Although our study lasted only one year, we could not detect a significant difference in the final visual outcomes.

Based on the literature, our results are inhomogeneous due to the different treatment protocols. In the DRCR.net Protocol T wherein the vitreous condition was not defined, the 1-year outcomes showed that the median number of IVA injections was 10, which is consistent with our results [5, 10]. In a retrospective study on non-vitrectomized and vitrectomized eyes receiving ranibizumab in a 6-month period, Chen et al. reported that ranibizumab had greater drug clearance in vitrectomized eyes, resulting in lower therapeutic levels. However, the follow-up period was relatively short compared to other studies [18]. In the DRCR.net Protocol I, an exploratory post-hoc assessment of the 3-year data of eyes with DME with and without prior vitrectomy that received ranibizumab and laser therapy revealed that improvement is slower, and the number of ranibizumab injections was significantly higher, in the vitrectomized group in the first year; however, there were no differences at the 3-year endpoint [20]. As these studies have inherent differences (e.g., examined intravitreal drug, distribution of the different vitreous condition eyes, etc.) from our study, a comparison with our trial cannot be conducted. Conversely, Tran et al. examined aflibercept therapy in vitrectomized eyes with DME [19]. Similar to our study, they prospectively assessed the number of IVA injections for 1 year and had no control group, only a PPV group. In contrast, their study involved only five monthly IVA injections followed by an unsimilar PRN regimen. The presence of any fluid was the criterion for re-treatment, and no BCVA and CSFT stability was considered unlike in protocol T and in our study. Their results revealed that the mean number of IVA injections was 9.3 ± 1.8 [19], which is comparable with the results in our PPV group (9.4 ± 2.1).

Regarding functional and anatomical outcomes after IVA injection, both vitrectomized and non-vitrectomized eyes showed significant BCVA gains and CSFT reductions at M6. Although non-vitrectomized eyes appeared to respond better to aflibercept overall, this observation does not confirm that an intact vitreous leads to superior outcomes, as the M6 results included refractory cases. When considering only the non-refractory eyes—those that continued IVA alone throughout the 1-year follow-up, which constituted our primary outcome analysis— we did not observe any significant differences in BCVA at any time point, and the only significant difference was in CSFT at M6, which was no longer present at M12. Within this non-refractory group, both vitrectomized and non-vitrectomized eyes demonstrated significant improvement in BCVA and CSFT from baseline to M12. However, changes between 6 and 12 months were not significant in either group, suggesting that aflibercept reaches its maximal therapeutic effect within the first 6 months, after which continued treatment primarily maintains, rather than further enhances, anatomical and functional outcomes. This pattern aligns with evidence from randomized clinical trials and real-world practice in DME, where the greatest improvements occur during the first 6 months of anti-VEGF therapy, followed by more modest gains thereafter [22–26].

It is important to note that baseline characteristics differed between groups. Vitrectomized eyes had more advanced DR and included fewer treatment-naïve cases (Table 1). Therefore, a multivariate ANCOVA was performed which revealed that these factors did not influence the M12 BCVA and CSFT results.

Although we followed the DRCR.net Protocol T regarding follow-ups and treatment, differences in study methodology exist, which can influence the direct comparison of functional and anatomical outcomes. In our study, we categorized patients based on the vitreous status, whereas Protocol T did not take this into account. Additionally, in the PRN phase, we performed DEX implantation instead of macular laser as treatment for refractory cases. Furthermore, in Protocol T, the subgroup of patients with a BL BCVA of < 69 letters (our mean BL BCVA was also < 69 letters in both groups) exhibited significant BCVA improvement at the 1-year follow-up, with a mean gain of 19.4 ± 11.1 letters [10]. If we compare this result with those of non-refractory cases treated with IVA injections alone in this study (7.0 ± 2.1 and 11.1 ± 2 letters in the PPV and non-PPV groups, respectively), they are inferior to those in Protocol T. Regarding retinal thickness, in Protocol T, the mean CSFT reduction after 1 year in the subgroup with a BL BCVA of < 69 letters was 212 ± 152 μm, which was comparable with our findings (182.5 ± 29.2 μm in the PPV group and 190.4 ± 26.6 μm in the non-PPV group) [10]. Despite similar anatomical changes, there was a greater functional improvement in Protocol T, which can be explained by differences in study methodology or BL characteristics [2, 10, 22, 23]. Compared with the results of Tren et al., who analyzed only vitrectomized eyes, they had worse anatomical outcomes, with a mean CSFT reduction of 108 μm. However, the functional outcomes were similar with ours, with a mean BCVA gain of 6 letters over 12 months [19].

Not all eyes with DME respond optimally to anti-VEGF therapy [1, 2]. Refractory DME is not uncommon, but there are several factors associated with it. Notably, there is no universally recognized definition, treatment protocol, or clinical marker of response to treatment [27]. In our study, the refractory criteria were based on the study of Busch et al. [27]. We found no significant difference between vitrectomized and non-vitrectomized eyes in the number of refractory cases that required DEX implantation after six IVA injections. Based on the collected data, we attempted to determine a predictive factor for the early identification of refractory cases. If the CSFT reduction at M1 was < 10.5%, that eye was expected to be refractory to IVA treatment. Altogether, our findings correlate with the existing clinical evidence that suggests that eyes demonstrating some (< 10% reduction in central retinal thickness) improvement after several injections of an anti-VEGF agent may be candidates for alternative therapies [8, 28]. However, there is no ideal timing on when to shift treatment, and no clear predictive factors that would assist in identifying patients who may experience delayed or late response to ongoing treatment with the same agent or who would benefit from switching have been identified [8].

Eyes that are refractory to anti-VEGF treatment may benefit from switching to intravitreal corticosteroids, especially DEX implants, early in the treatment course [2, 7]. Studies have established the efficacy and safety of DEX implants in eyes with DME refractory to conventional treatments regardless of the vitreous condition [27, 29]. In our study, all refractory cases received DEX implantation, and we examined the BCVA and CSFT 2 months after. We opted for this duration because the maximal BCVA gain and CSFT reduction manifested at 2 months after the first intravitreal DEX implantation in the CHAMPLAIN study [29]. In our trial, functional and anatomical improvements were noted, but only the reduction in CSFT was significant. This indicates that while DEX implants may effectively reduce retinal thickness in refractory cases, their impact on visual improvement remains limited as refractory eyes may have more negative prognostic BL characteristics restricting functional improvement [8].

Our study had several limitations. Our study had a small sample size and the BL characteristics were imbalanced. The latter reflects real-world conditions but complicates direct comparisons. For example, in terms of DR stage, there were more cases of proliferative DR in the PPV group, which can explain the differences between the two vitreous condition groups and the near significant difference in the mean BL BCVA in all eyes. However, balancing the characteristics would be difficult in a real-world study, similar to ours, because the need for PPV is generally justified in more serious cases. Prognostic OCT biomarkers (e.g., disorganization of the retinal inner layers (DRIL), ellipsoid zone disruption, external limiting membrane (ELM) disruption, hyperreflective foci) were not analyzed in this study; this is considered in the future because they can also restrict improvements. Moreover, we did not differentiate refractory cases according to the vitreous status after switching to DEX implants due to the small sample size (*n* = 13); this should be conducted in future studies with a larger sample size and longer follow-up period.

## Conclusion

In conclusion, aflibercept is a safe and effective treatment option for DME regardless of vitreous status with no significant difference in the number of IVA refractory eyes and IVA injections. Patients with DME treated with IVA injections showed significant functional and anatomical responses both in vitrectomized and non-vitrectomized eyes. Cases refractory to IVA treatment showed improvements in BCVA and CSFT following DEX implantation, with the anatomical outcomes being superior to the functional outcomes. Future studies with larger sample sizes are warranted to confirm our results and identify the optimal timing for shifting of treatment.

## Data Availability

Data and materials can be asked from the correspondence author by Email.

## Abbreviations

DME: Diabetic macular edema
CI-DME: Center-involved diabetic macular edema
DR: Diabetic retinopathy
VEGF: Vascular endothelial growth factor
DEX: Dexamethasone
BL: Baseline
PPV: Pars plana vitrectomy
IVA: Intravitreal aflibercept
SiO: Silicone oil
BCVA: Best-corrected visual acuity
CSFT: Central subfield thickness
SD-OCT: Spectral-domain optical coherence tomography
PRN: As needed
ANOVA: Analysis of variance
LSD: Least significant difference
ANCOVA: Multivariate analysis of covariance
ROC: Receiver operating characteristic
VA: Visual acuity
DRIL: Disorganization of the retinal inner layers
ELM: External limiting membrane
